# Rare Cause of Severe Mitral Regurgitation after TAVI: Case Report and Literature Review

**DOI:** 10.3390/medicina58040464

**Published:** 2022-03-23

**Authors:** Horațiu Moldovan, Bogdan-Ştefan Popescu, Elena Nechifor, Aida Badea, Irina Ciomaga, Claudia Nica, Ondin Zaharia, Daniela Gheorghiță, Marian Broască, Camelia Diaconu, Cătălina Parasca, Ovidiu Chioncel, Vlad Anton Iliescu

**Affiliations:** 1 Faculty of Medicine, Carol Davila University of Medicine and Pharmacy, 050474 Bucharest, Romania; ondin.zaharia@gmail.com (O.Z.); drcameliadiaconu@gmail.com (C.D.); ochioncel@yahoo.co.uk (O.C.); vladanton.iliescu@gmail.com (V.A.I.); 2 Bucharest Clinical Emergency Hospital, 014461 Bucharest, Romania; bogdan-stefan.popescu@outlook.com (B.-Ş.P.); aida.badea@sanador.ro (A.B.); bianca.nica@yahoo.com (C.N.); marian.broasca@gmail.com (M.B.); 3 Sanador Clinical Hospital, 011038 Bucharest, Romania; nechiforelena21@yahoo.com (E.N.); irina_ciomaga@yahoo.com (I.C.); 4 ”Prof. Dr. Theodor Burghele” Clinical Hospital, 061344 Bucharest, Romania; 5 Faculty of Materials Science and Engineering, Politehnica University of Bucharest, 060042 Bucharest, Romania; 6 “Prof. Dr. C.C. Iliescu” Institute for Cardiovascular Diseases, 022328 Bucharest, Romania; catalina.parasca@drd.umfcd.ro

**Keywords:** mitral regurgitation (MR), anterior mitral leaflet (AML) perforation, transcatheter aortic valve replacement (TAVR)

## Abstract

Pre-procedure mitral regurgitation (MR) is a frequent coexistent finding in patients undergoing transcatheter aortic valve replacement (TAVR), and most of them (up to 55%) experience a significant improvement in MR after the procedure. Although seldom described, mitral valve perforation after TAVR is a potentially serious complication that physicians should be aware of, as moderate or severe MR in TAVR recipients is associated with a high early mortality rate. We herein describe the case of a 65-year-old man presenting with worsening heart failure symptoms 5 months after TAVR due to an intraprocedural anterior mitral leaflet perforation and discuss the diagnostic process and therapeutic course of the case. Furthermore, we draw attention to the essential role of echocardiography in the management of TAVR procedures, taking into account its ability in detecting early complications, and emphasize the value of CT as a main determinant to predict long-term MR improvement after TAVR and to assess the potential candidates for double valve repair with percutaneous techniques.

## 1. Introduction

Mitral regurgitation (MR) of varying degrees frequently coexists with significant aortic stenosis (AS) [1,2,3,4]. This association involves an increased surgical risk and contributes to subsequently worse long-term clinical outcomes and higher morbidity in patients undergoing aortic valve replacement (AVR) [5,6,7,8,9]. The prevalence of moderate or severe MR in transcatheter aortic valve implantation (TAVI) recipients varies between 22% and 48%, as different series have reported [10,11,12,13,14,15,16,17,18]. As opposed to patients undergoing surgical AVR who can benefit from concomitant mitral valve repair or replacement, in the specific setting of TAVR, the MR is typically left untreated [2], although there is no doubt that preprocedural MR can potentially lead to left ventricular (LV) failure after the procedure [19], hence adversely influencing the prognosis in TAVR patients as well [4]. Furthermore, in the scenario of TAVR, the pathophysiologic mechanisms of postprocedural mitral valve malfunction also rely on the anatomical particularities of the mitral–aortic continuity. Being in close contact at the level of the left fibrous trigone, the mitral valve may be exposed to significant changes in both geometry and structure due to mechanical alteration of the aortic root, changes that can potentially result in functional MR or exacerbate the impairment of a pathological mitral valve [20]. Although seldom described, anterior mitral leaflet perforation after TAVR is a well-known, potentially life-threatening complication after TAVR [21], also entailing the risk of subsequent infective endocarditis (IE) [22,23,24,25,26,27]. Considering the widespread use of TAVR, which in recent years has been established at a rapid pace as the standard of care for the management of patients with symptomatic severe aortic stenosis and high or prohibitive risk for standard surgical treatment, awareness should be raised regarding the vulnerability of the mitral apparatus, the mechanisms of its impairment, and the potential complications that may occur during TAVR procedures, in order to develop better strategies to avoid, recognize, and manage those complications [20,28]. To be underlined is also the importance of multimodality imaging in the management of TAVR patients, not only to predict long-term MR improvement after TAVR, but also to select those patients who will benefit from a concomitant percutaneous repair procedure of the mitral valve (MitralClip or balloon expandable valves).

## 2. Case Report

**Figure 1:**
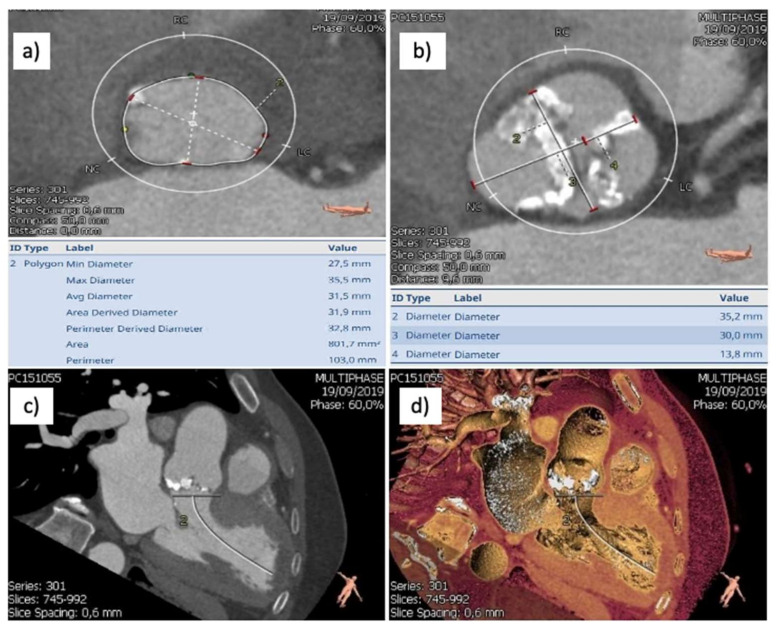
(**a**) Aortic annulus aspect on angio CT-large aortic annulus; (**b**) aortic valve aspect on angio CT-type 1 Sievers, left to right cusp fusion, severe asymmetric calcifications; (**c**,**d**) MDCT image showing significant NCS calcifications.

**Figure 2:**
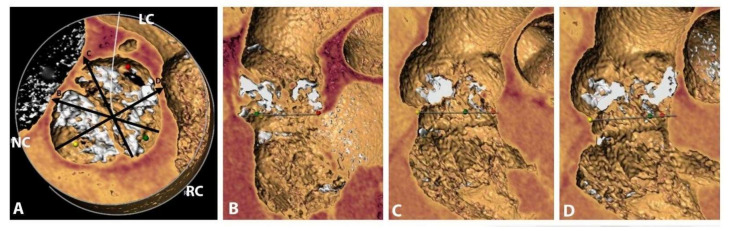
Aortic valve MDCT reconstruction: (**A**) Type 1 Sievers bicuspid aortic valve with severe asymmetric calcification and calcified raphe between left coronary cusp and right coronary cusp; (black arrows show section plan of images **B**–**D**); (**B**) Aortic root reconstruction—section plan through non-coronary cusp and right cusp with bulky asymmetric calcification towards aortic-mitral curtain; (**C**) Aortic root reconstruction—section plan through left-coronary cusp and right cusp with bulky asymmetric calcification towards aortic-mitral curtain; (**D**) Aortic root reconstruction—section plan through non-coronary cusp and calcified raphe.

A 65-year-old male patient presenting for NYHA class IV congestive heart failure symptoms was diagnosed after an initial evaluation with degenerative bicuspid aortic valve disease (low-flow low-gradient severe aortic stenosis, moderate aortic regurgitation) and concomitant moderate left ventricular systolic dysfunction. Comorbid conditions to be mentioned included left pneumonectomy followed by chemotherapy for squamous cell carcinoma, severe pulmonary hypertension, Child-Pugh class B non-viral hepatic cirrhosis, chronic kidney disease stage 3A with moderate normocytic anemia and a history of acute coronary syndrome with normal coronary arteries. Computer tomography revealed a heavily calcified (Ca score 2388) type-1 bicuspid aortic valve, a large aortic annulus (area 814 mm^2^, above manufacturers’ recommended maximum value) (Figure 1a,b) and significant aortic annular calcification, mainly distributed at the level of the non-coronary sinus (Figure 1c,d and Figure 2).

**Figure 3:**
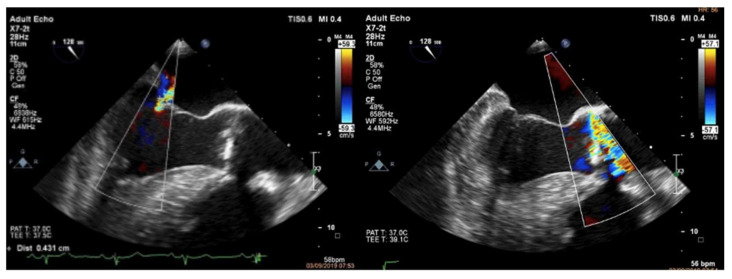
Preprocedural transesophageal echocardiography—moderate mitral regurgitation.

Despite the relatively young age and aortic bicuspid valve, the patient was deemed high risk for surgery due to his associated comorbidities and was referred for transcatheter aortic valve replacement (TAVR). After several days of medical treatment, informed consent was obtained, and the patient was placed under general anesthesia with invasive hemodynamic and transesophageal echocardiography monitoring. Preprocedural transesophageal echocardiography showed moderate mitral regurgitation (Figure 3).

Severe calcifications of type 1 bicuspid valve morphology and requirement of a 29-mm prosthesis led to the decision to predilate despite the presence of a large aortic annulus.

**Figure 4:**
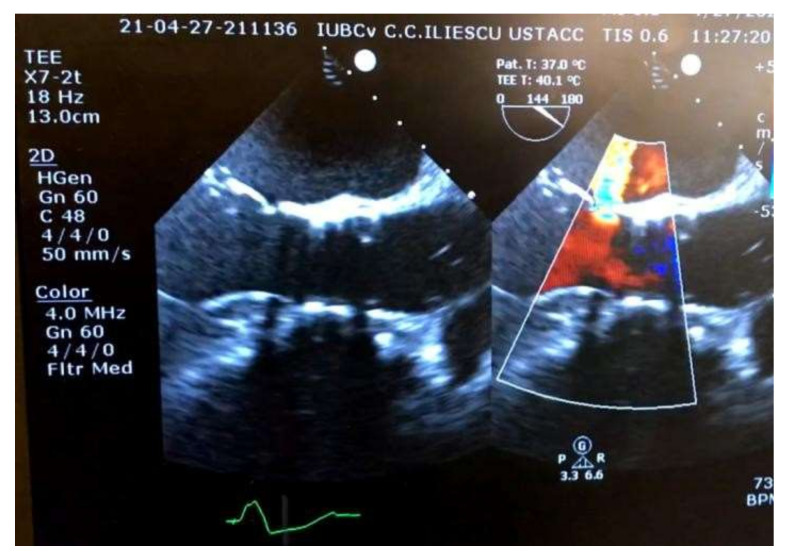
Intraprocedural transesophageal echocardiography—mitral regurgitation, AML perforation.

After predilatation with a No.25 balloon, intraoperative transesophageal echocardiography revealed an eccentric regurgitant jet at the base of the anterior mitral leaflet due to perforation in the medial side of the aortic–mitral curtain (Figure 4).

**Figure 5:**
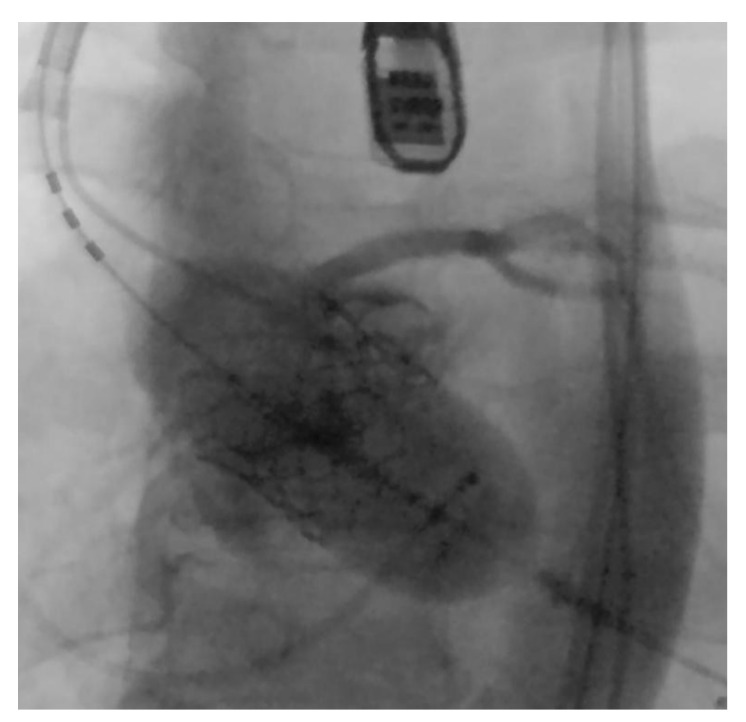
Valve implantation—intraprocedural fluoroscopy.

As the patient remained hemodynamically stable, the procedure continued with the successful implantation of a 29-mm Edwards SAPIEN-S3 valve (Edwards Lifesciences, Inc., Irvine, CA, USA) in the optimal position, deployed via the femoral approach. Fluoroscopy the revealed stable and optimal positioning of “locked-in-leaflet” aortic prosthesis, with a distorted stent frame due to folded bulky leaflet calcifications (Figure 5).

**Figure 6:**
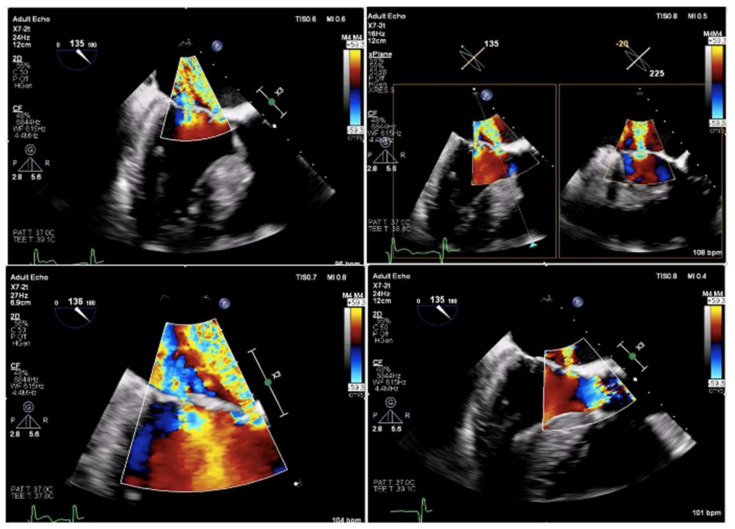
Intraoperative transesophageal echocardiography findings just after deployment of the transcatheter aortic valve showing anterior mitral leaflet perforation that caused an increase in the degree of MR.

Persistence of the regurgitant jet at the base of the anterior mitral leaflet presence precluded further post-dilatation despite the presence of a mild paravalvular leak (Figure 6).

**Figure 7:**
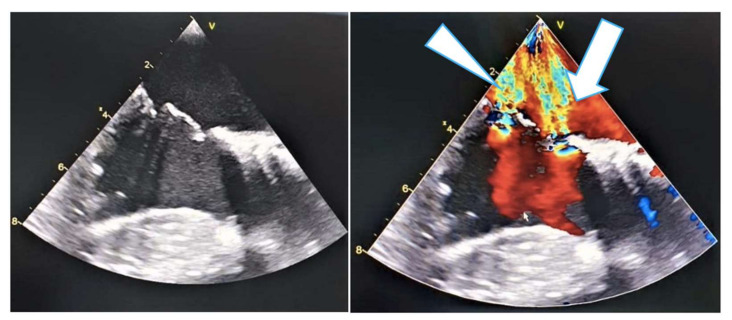
Intraoperative transesophageal echocardiographic evaluation of severe mitral regurgitation. Two regurgitation jets can be identified, one transvalvular (arrowhead) and a second through a perforation in the anterior mitral leaflet (arrow).

Postprocedural recovery was uneventful, allowing the patient to be discharged on the second postoperative day. Five months after the TAVR procedure, the patient presented with progressive worsening of heart failure symptoms. Transthoracic echocardiography revealed perforation of the anterior mitral leaflet causing severe mitral regurgitation (Figure 7); thus, the patient was scheduled for surgical mitral valve replacement. No clinical criteria for endocarditis were present at admission.

**Figure 8:**
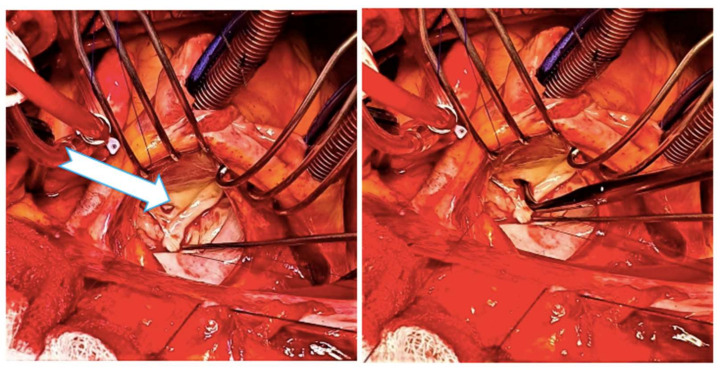
Intraoperative aspect of the perforated anterior mitral leaflet. The perforation could be clearly seen at the base of the AML (arrow).

**Figure 9:**
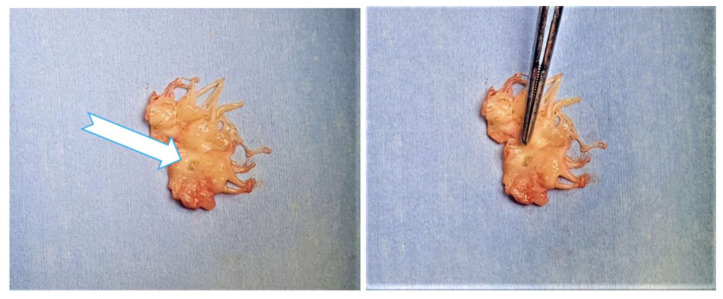
Excised anterior mitral leaflet, with a 5/10-mm perforation near its basal aspect (arrow).

An intraoperative 5/10-mm perforation could be observed at the base of the anterior mitral leaflet (Figure 8 and Figure 9). Despite a well-positioned aortic valve prosthesis, a couple of centimeters belonging to the bioprosthetic metal support’s proximal part came into contact with the anterior mitral leaflet, this being the causative mechanism of the leaflet perforation. The native mitral valve was excised, and a No.27 Hancock II biological prosthesis was implanted. The postoperative course was uneventful except for paroxysmal atrial fibrillation episodes, moderate transient thrombocytopenia and a slightly elevated serum creatinine level. The patient was discharged to the home ten days postoperatively. Predischarge transthoracic echocardiography showed correct function of the mitral bioprosthesis and no residual regurgitation. Similarly, at the one-month follow-up, the TTE did not show any evidence of HF, IE or deterioration of the transmitral gradient.

## 3. Discussion

We described the case of a patient presenting with progressive worsening of heart failure symptoms 5 months after TAVR with a 29-mm Edwards SAPIEN-S3 valve. Albeit rarely described, anterior mitral leaflet perforation after TAVR is a well-known complication considering the anatomy of the mitral–aortic curtain. AML perforation can lead to heart failure and infective endocarditis [29]. The impairment of the mitral apparatus can be imputable to several reasons, including: direct mechanical damage due to the incorrect (too low) deployment of the prosthesis [22,23]; the severe calcifications distributed at the level of the aortic sinuses; or the impingement of the mitral annular calcifications (MAC) by the expandable valve [29].

Secondary MR mechanisms encompass new-onset LBBB, LV dyssynchrony, and myocardial ischemia with papillary muscle dysfunction, cardiac tamponade, systolic anterior motion (SAM) of the mitral valve leaflet, and significant paravalvular leakage [30,31,32].

We can safely presume that the risk of mitral valve involvement is more likely with the CoreValve prosthesis (Medtronic, Minneapolis, Minnesota) than with the Edwards SAPIEN-S3 valve, the former having a larger component part extending into the LV outflow tract [30].

In our particular case, regardless of the optimal implantation of the valve, the AML perforation was most probably caused by the impingement of the significant aortic annular calcification block, mainly distributed at the level of the non-coronary sinus, toward the medial aspect of the mitral–aortic curtain, during the predilatation maneuver. Intraprocedural transesophageal echocardiography clearly demonstrated the mechanism of the increase of severity of MR (real-time feedback).

As TAVR is rapidly becoming a routine for the treatment of symptomatic severe AS in patients with a prohibitive surgical risk on account of its minimal invasiveness and comparable good short- and mid-term outcomes [31], careful attention should be paid to acute changes in the degree of MR, which have to be promptly assessed and adequately managed. Persistent significant or worsening MR following TAVR may be particularly important in patients who develop a significant paravalvular leak. In such patients, a second step intervention should be considered by a multidisciplinary heart team—either a percutaneous edge-to-edge mitral valve repair (MitraClip) or a paravalvular leak closure (or both) [4]. Consequently, precise characterization of both the aortic valve and the mitral apparatus has to be warranted before TAVR, aiming to assess the potential candidates for double valve repair using percutaneous techniques.

Lastly, we would like to highlight the importance of detailed CT interpretation not only of the aortic valve, but also regarding the anatomy of the mitral apparatus and especially calcifications, since calcification of the leaflets and the annulus predict the persistence of MR and the increase in cardiac mortality [2]. A thorough evaluation of these imaging parameters can become clinically relevant when planning a therapeutic strategy for high surgical risk patients with multivalvular disease [32,33,34,35,36].

Our case emphasizes, therefore, not only the inherent risk of AML perforation following TAVR, but also that meticulous patient evaluation and selection by means of multimodality imaging is the sine qua non of the catheter-based aortic valve replacement.

## 4. Conclusions

The rapid change in paradigm from the standard surgical treatment of aortic stenosis to percutaneous options unveils the importance of exhaustively understanding the anatomical, functional, and clinical implications of TAVR as a therapeutic entity. The increase in severity grade of MR during TAVR can be attributable to various and complicated mechanisms and should be promptly assessed and comprehensively managed in accordance with its precise mechanisms. Further prognostically relevant factors should be evaluated in future clinical trials in order to optimize treatment and improve long-term prognosis in patients with concomitant MR undergoing TAVR, as long as the impact of moderate or severe concomitant MR on clinical outcomes has not been extensively studied so far, given that severe MR has been considered an exclusion criteria for TAVR.

## Data Availability

The data presented in this study are available on reasonable request from the corresponding author.

## Abbreviations and Acronyms

AS	aortic stenosis
IE	infective endocarditis
HF	heart failure
LBBB	left bundle-branch block
LV	left ventricular
MDCT	multidetector computed tomography
MR	mitral regurgitation
TAVR	transcatheter aortic valve replacement
TEE	transesophageal echocardiography
TTE	transthoracic echocardiography
